# Comparison of Partial Meniscectomy With Meniscal Repair With Respect to Functional Outcome

**DOI:** 10.7759/cureus.55643

**Published:** 2024-03-06

**Authors:** Luqman Khan, Rao E Hassan, Haroon Zaid, Zeeshan Haider, Ubaid Ullah, Adnan Ahmad, Imtiaz Rehman, Vemparala Priyatha, Abdul Hameed Khan, Yaseen Ahmad

**Affiliations:** 1 Orthopaedics and Trauma, Khyber Teaching Hospital MTI, Peshawar, PAK; 2 Internal Medicine, All India Institute of Medical Sciences, Bhubaneswar, Bhubaneswar, IND; 3 Internal Medicine, Combined Military Hospital, Peshawar, PAK; 4 General Surgery, Ayub Teaching Hospital, Abbottabad, PAK

**Keywords:** meniscal tear, good functional outcome, partial meniscectomy, meniscal repair, anterior cruciate ligament injury

## Abstract

Introduction

Meniscus tear is a commonly encountered sports-related injury requiring surgical intervention due to knee mobility dysfunction and discomfort. Previously, it has been thought that these are non-functional vestigial structures and they used to be excised commonly. Recent studies have shown that meniscal repair gives superior results when compared with partial meniscectomy.

Methods

This quasi-experimental study was conducted at the Orthopedics Department, Khyber Teaching Hospital, Peshawar, Pakistan. A total of 92 patients of both genders with meniscal injuries were included. Forty-six of them underwent meniscal repair (Group A), and 46 underwent partial meniscectomy (Group B). Functional outcome was noted after 12 weeks and recorded.

Results

The age range was from 18 to 50 years with a mean of 28.630±6.64 years in Group A and 29.630±8.12 years in Group B. Functional outcome was excellent in 44 (95.7%) patients who underwent meniscal repair as compared to 23 (50%) patients who underwent partial meniscectomy (P= 0.000).

Conclusion

It is concluded that meniscal repair should be pursued over partial meniscectomy when surgically treating meniscal tears.

## Introduction

A meniscus tear is a common sports-related knee injury that often requires surgery due to knee mobility dysfunction and discomfort [1]. Previously, these tears were considered non-functional vestigial structures and were commonly excised [1]. With increased participation in sports among the general population and the use of advanced diagnostic methods, the documented incidence of meniscal tears is approximately 60 tears per 100,000 persons, although this incidence is thought to be underestimated. Patients with such injuries experience accelerated cartilage wear, putting them at risk of early-onset osteoarthritis and reduced functionality. Interestingly, three out of every four patients diagnosed with osteoarthritis have some type of meniscus-related injury [2].

Treatment for a meniscal tear can be conservative or surgical depending on the grade of the injury. Surgical interventions include meniscal repair or meniscectomy and are necessary for most tears [2]. Initially performed by Annandale in 1885 using an open technique, several arthroscopic techniques have since been developed and are in use. Total meniscectomy was previously considered the treatment of choice until the late 1970s [2]. However, recent studies have shown multiple drawbacks of meniscectomy, including flattening of the femoral condyle and decreased joint space leading to osteoarthritis [3]. Menisci are now recognized as vital structures, and their preservation is encouraged. Saving the menisci, where possible, has been shown to decrease the incidence of osteoarthritis and knee instability in post-surgical patients [4].

Despite better short-term outcomes, simplicity, and shorter operative time, partial meniscectomy is still widely performed [5,6]. In contrast, meniscal repair, initially performed arthroscopically by Ikeuchi in 1969, has been shown to offer superior advantages over partial meniscectomy in terms of patient outcomes [5]. Paxton et al. demonstrated excellent functional outcomes (100%) with meniscal repair compared to partial meniscectomy (54.2%) [7].

The choice between repair and partial meniscectomy as treatment options is based on the belief that repair will result in superior outcomes. As no such study has been conducted in the study population before, it was planned to compare the functional outcome of meniscal repair and partial meniscectomy in patients with meniscal tears, with or without anterior cruciate ligament (ACL) injury.

## Materials and methods

The Department of Orthopedics at Khyber Teaching Hospital in Peshawar, Pakistan, conducted a quasi-experimental study over a six-month period (December 2022-June 2023). The sample size was calculated using the OpenEpi calculator, with an expected frequency of poor functional outcomes at 0% versus 15.5%, respectively [7]. The estimated sample size was 92, with 46 patients assigned to the meniscal repair group (Group A) and 46 patients to the partial meniscectomy group (Group B). Convenience sampling was employed for the recruitment of individuals into the study.

Patients aged 18-50 years, of either gender, with grade three or four meniscal tears with or without ACL injury were included. Patients with third- or fourth-grade chondromalacia in any compartment, those who had previously undergone knee surgery, and patients undergoing concomitant surgery during ACL reconstruction for other injuries were excluded. Additionally, meniscus tears considered inappropriate for repair, whether due to location, size, or tissue quality, were excluded from the study.

Functional outcome was assessed using the Lysholm-Tegner Knee Scoring Scale [8] 12 weeks post procedure, with scores categorized as follows: Excellent (90-100), Good (65-90), and Poor (≤64).

Patients who met the inclusion criteria were enrolled in the study following approval from the local ethical committee (Khyber Medical College Institutional Research and Ethical Review Board approval no. 846/DME/KMC). Subsequently, they received a comprehensive explanation regarding their participation in the study and provided written consent, which delineated the risks and benefits involved. Arthroscopy was performed via standard conventional anteromedial and anterolateral arthroscopic ports. The meniscectomy procedure involved utilizing a meniscal biter instrument to trim the edge of the torn meniscus, aligning it with the healthy area for consistency. Following this step, an arthroscopic shaver meticulously smoothed the meniscal edge while simultaneously removing any remaining tissue fragments from the joint. Meniscal repairs were carried out using the TightRope® technique by the inside-out method. In cases where necessary, standard ACL reconstruction was performed using the attachable button system (ABS) and EndoButton (Smith & Nephew, Mokena, IL).

Post surgery, patients were instructed to avoid bearing weight for a period of two weeks, followed by two months of partial weight-bearing. Functional outcomes (categorized as Excellent, Good, and Poor) were evaluated at the 12-week mark and recorded using a specially designed proforma.

Data analysis was conducted using Statistical Product and Service Solutions (SPSS, version 24.0; IBM Corp., Armonk, NY). Quantitative analysis entailed calculating frequencies and percentages for categorical variables such as gender and the classifications of Excellent, Good, and Poor. Means ± standard deviations (SD) were utilized for quantitative variables such as age and duration of injury. Functional outcomes between the two groups were compared using a chi-square test, with statistical significance set at p ≤ 0.05. Statistical significance was determined through a post-stratification chi-square test conducted on both groups, with a significance level set at p ≤ 0.05.

## Results

The mean age of participants in Group A was 28.63 ± 6.64 years, with a mean injury duration of 3.00 ± 1.15 months. In Group B, the mean age was 29.63 ± 8.12 years, and the mean injury duration was 3.65 ± 1.32 months.

Male gender predominated in both groups (Table 1). Excellent functional outcomes were observed in 44 (95.7%) patients in Group A compared to 23 (50%) patients in Group B, with good outcomes noted in one (2.2%) versus 15 (32.6%) patients, and poor outcomes in one (2.2%) versus eight (17.4%) patients, respectively (p = 0.000) (Table 2).

**Table 1:** Frequency and percentage of gender in both groups

Gender	Group A (n=46)	Group B (n=46)
Male	29 (63%)	24 (52.2%)
Female	17 (37%)	22 (47.8%)
Total	46 (100%)	46 (100%)

**Table 2:** Comparison of functional outcome in both groups The chi-square test was used with a p-value ≤ 0.05 significant.

Functional outcome	Group A (n=46)	Group B (n=46)	P-value
Excellent	44 (95.7%)	23 (50%)	0.000
Good	1 (2.2%)	15 (32.6%)	
Poor	1 (2.2%)	8 (17.4%)	
Total	46 (100%)	46 (100%)	

Stratification of functional outcomes in both groups by age (Table 3), gender (Table 4), and injury duration (Table 5) is presented.

**Table 3:** Stratification of functional outcome with respect to age in both groups The chi-square test was used with a p-value ≤ 0.05 significant.

For age 18-30 years				
Group	Functional outcome			P-value
	Excellent	Good	Poor	
A	35 (97.2%)	1 (2.8%)	0 (0%)	0.000
B	18 (54.5%)	12 (36.4%)	3 (9.1%)	
For age 31-50 years				
Group	Functional outcome			P-value
	Excellent	Good	Poor	
A	9 (90%)	0 (0%)	1 (10%)	0.038
B	5 (38.5%)	3 (23.1%)	5 (38.5%)	

**Table 4:** Stratification of functional outcome with respect to gender in both groups The chi-square test was used with a p-value ≤ 0.05 significant.

For Male				
Group	Functional outcome			P-value
	Excellent	Good	Poor	
A	28 (96.6%)	0 (0%)	1 (3.4%)	0.000
B	12 (50%)	8 (33.3%)	4 (16.7%)	
For Female				
Group	Functional outcome			P-value
	Excellent	Good	Poor	
A	16 (94.1%)	1 (5.9%)	0 (0%)	0.012
B	11 (50%)	7 (31.8%)	4 (18.2%)	

**Table 5:** Stratification of functional outcome with respect to duration of injury in both groups The chi-square test was used with a p-value ≤ 0.05 significant.

For 1-3 months				
Group	Functional outcome			P-value
	Excellent	Good	Poor	
A	31 (100%)	0 (0%)	0 (0%)	1.000
B	20 (100%)	0 (0%)	0 (0%)	
For >3 months				
Group	Functional outcome			P-value
	Excellent	Good	Poor	
A	13 (86.7%)	1 (6.7%)	1 (6.7%)	0.000
B	3 (11.5%)	15 (57.7%)	8 (30.8%)	

## Discussion

In our study, we observed excellent functional outcomes in 44 (95.7%) patients in Group A compared to 23 (50%) patients in Group B. Good outcomes were noted in one (2.2%) versus 15 (32.6%) patients, while poor outcomes were seen in one (2.2%) versus eight (17.4%) patients (p = 0.000). Paxton et al. demonstrated superior functional outcomes (100%) following meniscal repair compared to those observed after partial meniscectomy (54.2%). Good outcomes were 0% versus 26.8%, and poor outcomes were 0% versus 15.5% [7].

The re-operation rate in partial meniscectomy is markedly lower in contrast to meniscal repair. Repairs, combined with anterior cruciate ligament repair (ACLR), show a reduced re-operation rate compared to isolated meniscal repairs. While partial meniscectomy is associated with a reduced risk of early-onset osteoarthritis in comparison to total meniscectomy, it remains unclear whether meniscal repair effectively mitigates these developments. However, compelling evidence indicates superior imaging and quality of life outcomes linked with meniscal repair over a prolonged period [9,10]. Although the rates of re-operation after meniscal repair are high (i.e., 23%), and along with concomitant ACLR is 13%, compared to a 4% re-operation rate after a partial meniscectomy, it can be deemed acceptable as there is a potential for long-term benefits in terms of functionality [7].

It is important to acknowledge that, although the potential benefits of meniscal repair are not conclusively established, the current literature provides only modest support for this idea. After meniscal repair, there is an increased likelihood of re-operation for the medial meniscus compared to the lateral one. This is possibly due to its stronger adherence to the tibial head, its greater capacity for bearing mechanical loads, and the laxity it experiences after ACLR, which exposes the medial meniscus to greater stress [11,12].

Meniscal repair can benefit from certain modifications during the procedure to enhance its efficacy. These modifications can include concomitant ACLR [13-15], iatrogenically damaging the surrounding synovium [16], leaving a fibrin clot in the repair site [17,18], introducing a microfracture in the notch [19], and using platelet-rich plasma inside the joint after the procedure [20].

There is a scarcity of comprehensive data concerning the prolonged effects of partial meniscectomy and meniscal repair. The International Knee Documentation Committee (IKDC) score and Fairbank grading are frequently employed as standard measures to evaluate the extent of degenerative alterations radiologically [21]. Rockborn et al. [22] observed a statistically significant difference in radiographic results at the seven-year follow-up, which was not apparent in the final assessment after 13 years. Repair patients exhibited a 16% lower incidence of mild degeneration (Fairbank grade 1) compared to meniscectomy patients, along with a 23% lower incidence of moderate degeneration (Fairbank grade 2) between the two groups. Additionally, joint space reduction was more prevalent following meniscectomy compared to repair [22].

Our study has certain limitations. The study's use of convenience sampling may introduce selection bias. Exclusion criteria, such as excluding patients with chondromalacia or prior knee surgeries, may restrict applicability to broader populations. Being a single-center study, results may not generalize to other settings. Additionally, ethnic and socioeconomic factors may affect applicability. These limitations warrant cautious interpretation of findings and consideration of the broader context in clinical decision-making.

In summary, while partial meniscectomy generally has lower re-operation rates compared to meniscal repair, combining meniscal repair with ACL reconstruction yields lower re-operation rates with favorable long-term outcomes. Factors contributing to higher re-operation rates in meniscal repairs include biomechanical loads and residual laxity post-ACLR. Considering the potential short-term and long-term advantages of meniscal repair for joint health, despite the likelihood of increased re-operation rates, it is reasonable to prioritize this procedure over partial meniscectomy to achieve improved functional outcomes. However, it is important to note that not all meniscal injuries are suitable for repair. Therefore, prioritizing meniscal repair whenever possible is crucial for maximizing the long-term function of the knee, as it preserves its original structure and functionality.

## Conclusions

We can conclude that meniscal repair should be favored over partial meniscectomy when surgically addressing meniscal tears, as it evidently results in better preservation of function. While partial removal may be necessary in certain cases, prioritizing the repair of torn menisci seems to offer patients the highest likelihood of returning to normal activity levels and sustaining knee health. Further research is required to refine techniques for successful repair and identify optimal surgical candidates. Nevertheless, our study provides compelling evidence that meniscal repair yields excellent functional outcomes in the majority of patients and should be the preferred surgical approach whenever possible.
